# Regulation of HSVtk gene by endogenous microRNA-122a in liver cell lines as suicide gene therapy 

**Published:** 2017

**Authors:** Maryam Ghanbari, Esmaeil Saberfar, Zahra Goodarzi, Hadi Lashini, Sahar Ghanbari, Mojtaba Karamimanesh, Kazem Baesi

**Affiliations:** 1 *Faculty of Advanced Sciences and Technology, Pharmaceutical Sciences Branch, Islamic Azad University (IAUPS), Tehran, Iran*; 2 *Researches and Development Department, Bayerpaul Group, Tehran, Iran*; 3 *Applied Virology Research Center, Baqyatallah University of Medical Sciences, Tehran, Iran*; 4 *Environmental Health Expert Center, Fasa University of Medical Sciences, Fasa, Fars, Iran*; 5 *Hepatitis and AIDS Department, Pasteur Institute of Iran, Tehran, Iran*

**Keywords:** microRNA regulation, miR-122a, HSVtk gene, ganciclovir, cancer therapy

## Abstract

**Aim:**

Here, we use miR-122a that exhibits liver-specific expression for developing a post-transcriptional regulative system mediated by microRNAs.

**Background:**

Gene therapy with adenovirus (Ad) vectors that express herpes simplex virus thymidine kinase (HSVtk) and ganciclovir (GCV) have been suggested as a therapeutic strategy to cancer. However, Ad vectors injected into tumors are dispersed into the systemic circulation and transduce liver cells, resulting in severe hepatotoxicity. To be effective, the delivery and expression of suicide genes to cancer treatment ought to be specific to tumor cells, and avoid death of healthy cells. Researchers have demonstrated that expression of transgene could be suppressed in healthy cells with use of vectors that are reactive to microRNA regulation.

**Methods:**

We constructed an Ad vector carrying four tandem copies of target sequences of miR-122a that were incorporated into 3'-UTR of HSVtk gene. The expression level of miR-122a was quantified in HepG2 and Huh7 cell lines.

**Results:**

Quantitative RT- PCR analysis demonstrated that Huh7 cells express large amounts of miR-122a compared to HepG2 cells. The viability of Huh7 cells and HepG2 cells after infection by Ad-tk-122aT vector was 83% and 23.5%, respectively. The viability of Huh7 cells was not reduced in the presence of GCV after infection by Ad-tk-122a vector. In contrast, cytotoxicity of HSV-tk/GCV was similar in Huh7 cells and HepG2 cells by Ad-tk vector, with 35.3% and 27% viability, respectively.

**Conclusion:**

Inclusion of the miR-122a target sequences in the HSVtk expression cassette yielded a feasible strategy for reducing cytotoxicity of suicide gene in a liver cell line with high miR-122a expression

## Introduction

 Of various approaches to cancer gene therapy, suicide cancer gene treatments using the herpes simplex virus thymidine kinase (HSVtk) gene and ganciclovir (GCV) have been demonstrated in clinical and preclinical researches (1, 2). The therapeutic effect of the HSVtk/GCV consists of transduction of the HSVtk gene in tumor cells and change of nontoxic GCV to highly toxic phosphorylated GCV by HSVtk gene. Following that, phosphorylated GCV inhibits DNA replication and causes cell death. Several gene delivery systems, including retrovirus vectors, adenovirus vectors, and liposomes, have been used in HSVtk/GCV gene therapy. Adenovirus vectors have several advantages over other viral and nonviral vectors, because they have high transduction efficacy and can be grown to high titers (3, 4).

Regulation of virus host range is of particular importance. For best therapeutic effects, gene therapy vehicles should target cancer cells while avoiding other organs and normal cells (5, 6). Suicide gene therapy is associated with hepatic damage, because Ad vectors have high tropism to liver cells. In order to increase the safety and efficacy of suicide gene therapy by HSVtk gene that is transduced by Ad vectors, unwanted effects caused by Ad vector in the liver should be be diminished, without reducing transgene expression in the tumor cells. Lately, researchers have focused on post-transcriptional regulatory systems mediated by microRNAs (miRNAs) (7, 8). MicroRNAs are noncoding small RNAs that contribute to the regulation of their cognate target genes, usually by incomplete base-pairing with the 3′-untranslated region (UTR) of the target mRNA, which results in cleavage/degradation of the mRNA and translational repression (8, 9).

 Insertion of miRNA target sequences into the 3′-UTR of a gene decreases the expression level of the gene, with the amount of reduction dependent on the cellular expression levels of the miRNA (8, 10, 11). Because miR-122a is highly expressed in liver cells, we hypothesized that insertion of sequences complementary to miR-122a into the 3′-UTR of HSVtk gene in Ad vectors will reduce HSVtk expression in liver cell without affecting HSVtk expression in cancer cells (12).

To test this hypothesis, we constructed an Ad vector containing four tandem copies of miRNA-binding sites for miR- 122a in 3′-UTR of HSVtk gene and used the hepatoma cell lines, Huh7 and HepG2, as models for suicide gene therapy with this Ad vector. We showed that endogenous miR-122a regulated HSVtk expression in Huh7 cell line with high level of endogenous miR-122a in comparison to HepG2 cell line with low level of endogenous miR-122a. 

## Methods

Fetal bovine serum (FBS) and Dulbecco’s modified Eagle’s medium (DMEM) were purchased from Gibco. The human hepatomata cell lines HepG2 and Huh7, and human embryonic kidney cell line AD-293 were obtained from the Groningen research institute of pharmacy (Groningen, The Netherlands). The replication-defective recombinant adenovirus vector, AdEasy™ Adenoviral Vector System, was obtained from Agilent Technologies.

TriPure Isolation Reagent was purchased from Roche. Universal cDNA Synthesis kit, LNA™-enhanced microRNA qPCR primer sets and SYBR® Green Master Mix kits were purchased from Exiqon. MTT Assay Kit was purchased from Promega. All experiments were performed at least three times and their average values are reported here. 

**Cell culture**

The human hepatomata cell lines HepG2 and Huh7, and human embryonic kidney cell line AD-293 were grown in high-glucose Dulbecco's modified Eagle's medium supplemented with 2 mM l-glutamine, 100 U of penicillin/mL, 100 μg of streptomycin/mL, and 10% fetal bovine serum. Cells were grown in 25 cm2 polystyrene tissue culture flasks in a humidified atmosphere of 5% CO2 at 37 ◦C.

**Plasmids and Ad vectors**

Recombinant adenovirus vector was generated from adenovirus type 5, and the E1 and E3 regions were deleted to prevent virus replication. The pSELECT-zeo-HSV1tk was purchased from Invivogen Company. We amplified tk gene with forward (F XhoI: 5- AATCTCGAGATGGCCTCGTACCCCGGCCA -3) and reverse (R EcoRI: 5-CCGGAATTCTTACATCTCACGGGCAAACG-3) primers containing Xho1 and EcoR1 site, respectively, in a PCR from pSELECT-zeo-HSV1tk. Briefly, a 1.7-kb XhoI/EcoRI fragment was purified and subcloned into pAd track shuttle, to form the pAd track-tk shuttle. Ad vectors containing miR-122a target sequences were established by in vitro ligation. A pAd track-tk/122a shuttle plasmid with four tandem copies of perfectly complementary sequences to miR-122a in the 3′-UTR of the HSVtk expression cassette, was established as follows: A EcoRI/BamHI fragment of miR-122a target sequences (5′- AATTCACAAACACCATTGTCACACTCCACAGCACAAACACCATTGTCACACTCCATTCGAAACAAACACCATTGTCACACTCCAGGACACAAACACCATTGTCACACTCCAA -3) (perfect complementary sequences of miR-122a are underlined) was ligated to pAd track-tk, resulting in pAd track-tk/122a. For generating Ad vectors (Ad, Ad-tk-122aT, and Ad-tk), the resultant plasmids were linearized by digesting with restriction endonuclease PmeI, and were subsequently co-transformed into E. coli (BJ5183 cells) with the pAdEasy-1 adenoviral backbone plasmid. Recombinants were selected for kanamycin resistance, and recombination was confirmed by restriction endonuclease analyses. Finally, the linearized recombinant plasmids were transfected into AD-293 cells with FuGENE® HD Transfection Reagent (Promega, USA), in accordance with the manufacturer’s instructions. Biological titers were measured using the Adeno-X rapid titer kit (Clontech, Mountain View, CA) according to the manufacturer’s instructions.

**miRNA expression analysis**

Total RNA was extracted from HepG2 and Huh7 cell lines using TriPure Isolation Reagent (Roche) following the manufacturer's protocol. Residual DNA was removed with use of RNase-free DNase I (Fermentase) following the manufacturer's protocol. To quantify miR-122a expression, real-time PCR was performed using the TaqMan miRNA assay kit for miR-122a by the Applied Biosystems 7500 Sequence Detection System (Applied Biosystems). The expression of miRNA was defined from the threshold cycle (Ct), and relative expression levels were calculated after normalizing with reference to expression of U6 small nuclear RNA.

**In vitro gene expression analysis**

HepG2 and Huh7 cells were seeded at 1000 cells/well on 96-well plates. On the following day, the cells were infected with Ad, Ad-tk and Ad-tk-122aT for 1.5 hours at 400 VP/cell. Following 24-hour incubation, 20µL of GCV was added to cells. After 3 days, the viability of cells was measured using a MTT Assay Kit (Promega). Briefly, MTT 10.0 µL (5.0 mg/mL) was added to each well and the cells were incubated at 37 ◦C for 4 hours. Then, the MTT-containing medium was removed and the formazan crystals formed by living cells were dissolved in 200.0 µL DMSO. Absorbance at 570 nm was determined by a microplate reader (ELx800™, BioTek, Winooski, VT) at test and reference wavelengths of 570 nm (13).

**Statistical analysis**

For calculation of miR-122a expression fold change, the expression level of miR-122a in each cell line was normalized to that of U6 snRNA, as the internal control. Then, miR-122a expression in Huh7 cell line was compared to the HepG2 cell line (2^-ΔΔCT).

 We used samples t-test to calculate statistical significance and data were considered statistically significant if P-values < 0.05. 

## Results

**Establishment of an Adeno vector that carry tandem copies of miR-122a-target sequences**

To test the effect of endogeneous miR-122a on transgene expression in hepatic cells, Ad vector was constructed containing miRNA-binding sites for miR- 122a (9). HSVtk gene was inserted into the XhoI and EcoRI sites of the Ad vector. Then, four tandem copies of sequences with perfect complementarity to miR-122a were inserted into the EcoRI and BamHI sites of the Ad-tk vector. 

**Figure 1:**
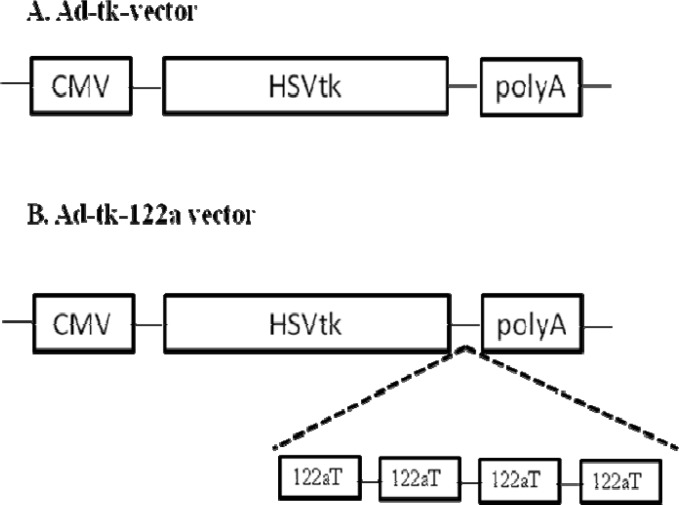
Schematic drawing of Ad vectors used in this study. (A) Ad-tk, (B) Ad-tk-122aT. CMV: CMV promoter; HSVtk: herpes simplex virus thymidine kinase gene; polyA: synthetic poly A; 122aT: miR-122a target sequences

Fig. 1B shows that Ad-tk-122aT vector carries HSVtk gene with four tandem copies of miR-122a complementary target sequences in its 3′-UTR. Ad-tk vector containing no miRNA target sequences was used as the control vector in this study (Fig. 1A).

**Endogenous level of miR-122a in Huh7 and HepG2 cell lines**

**Figure 2:**
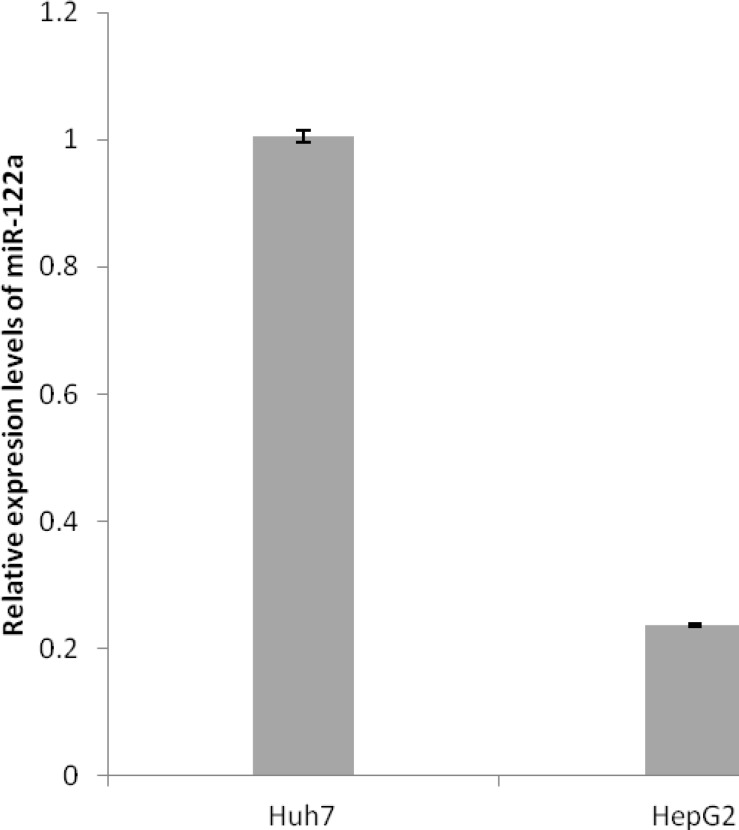
Relative expression levels of miR-122a in Huh7 and HepG2 cells as assessed by quantitative reverse transcriptase-PCR. The ratios of expression levels of miR-122a to U6 are shown as relative miR-122a expression levels. Data are presented as mean ± S.E. (n = 3)

miR-122a is highly expressed in the liver. Expression level of miR-122a in two human hepatoma cell lines, Huh7 and HepG2, were evaluated by quantitative RT- PCR. Quantitative RT- PCR analysis demonstrated that Huh7 cells express higher expression level of miR-122a compared to HepG2 cells (P = 0.000; Fig. 2). 

**Regulation of HSVtk gene in liver cell lines by miR-122a**

**Figure 3:**
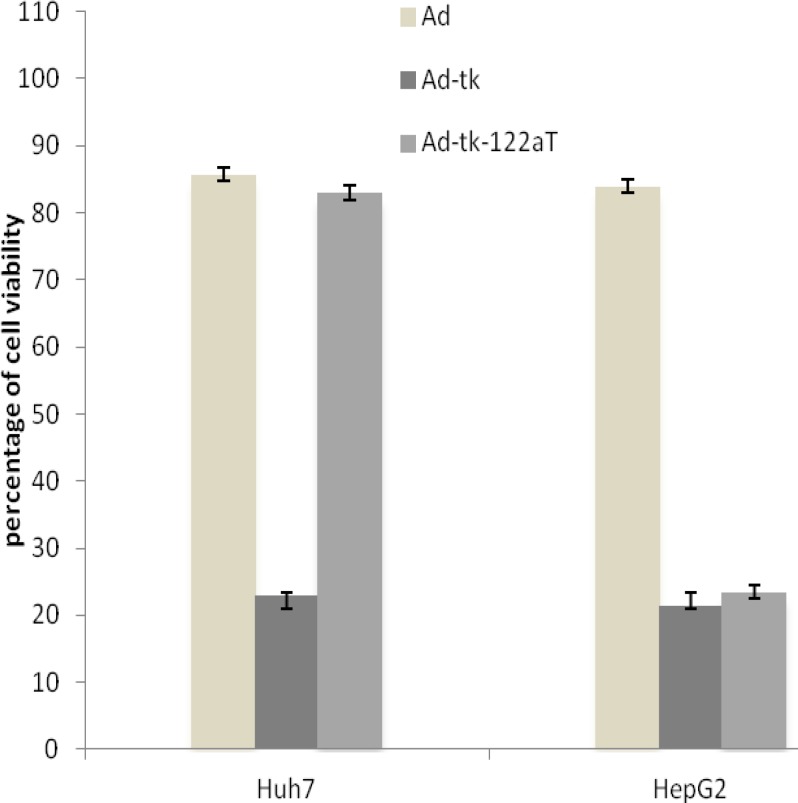
Cytotoxicity of HSVtk/GCV in Huh7 and HepG2 cells transfected by different Ad vectors (Ad, Ad-tk and Ad-tk-122aT). Cytotoxicity was measured by MTT assay kit. Ad: adenovector; Ad-tk: adenovector with herpes simplex virus thymidine kinase gene; Ad-tk-122aT: adenovector with miR-122a target sequences in3′-UTR region of herpes simplex virus thymidine kinase gene. Data are presented as mean ± SE (n = 3

We evaluated the Ad vector containing the miR- 122a target sequences for HSVtk/GCV suicide gene therapy in treating cancer. Ad-tk-122aT vector was constructed (Fig. 1B), and the cytotoxicity effect of HSVtkgene was measured in Huh7 and HepG2 cells that express various levels of miR-122a. Huh7 and HepG2cells were infected with Ad, Ad-tk and Ad-tk-122aT vectors at 400 VP/cell, and exposed to 20 µL/mL of GCV for 3 days. The viability of cells in each group was measured by MTT assay. As shown in Fig.3, HepG2 cells, but not Huh7 cells, were sensitive to GCV after infection with Ad-tk-122aT. The viability of Huh7 cells and HepG2 cells after infection with Ad-tk-122aT vector was 83% and 23.5%, respectively (P = 0.003; Fig. 3). The cytotoxicity effect of HSVtk/GCV was reduced in Huh7 cells infected with Ad-tk-122aT compared to that of HepG2 cells. This effect was likely caused by the higher expression levels of miR-122a in Huh7 cells. In contrast, the cytotoxicity of HSV-tk/GCV was similar in Huh7 cells and HepG2 cells infected with Ad-tk vector, with 35.3% and 27% viability, respectively (P = 0.796; Fig. 3). No cytotoxicity effect was demonstrated in either cell type after infection with the Ad vector (P = 0.862; Fig. 3).

## Discussion

The potential of HSVtk/GCV suicide gene therapy by Ad vectors has been widely reported (4). However, an important problem of this kind of therapy is the toxicity of the suicide gene to healthy tissue. So, regulation of suicide gene expression is required to increase the efficacy and safety of gene therapy (5, 9). Our study was conducted to evaluate the efficacy of using a miRNA-regulated transgene expression network for HSVtk gene in hepatoma cell lines infected with Ad vectors. Much consideration has been focused on using microRNAs to control gene expression. miRNA regulation has several advantages over transcriptional targeting, and a recent strategy consists of engineering of the vector that contains miRNA target (miRT) sequences which can be identified and regulated by cellular miRNAs (5, 9, 14). Because of the highly differential expression of many miRNAs between normal and malignant tissues, cellular miRNAs have been used to target gene therapy vectors with use of tumor suppressor miRNAs which are down-regulated in cancer cells (8, 15-17). Thus, insertion of tumor suppressor target sequences into a vector can limit the expression of transgene in normal cells, while the expression of transgene remain in tumors that lack these miRNAs; so, targeting by microRNAs could reduce toxicity to normal tissues and provide a new way of giving specificity to cancer tissues (4, 18, 19). The miR-122a is a liver specific miRNA, and down-regulated in liver cancer cells (12). Based on these detections, four tandem copies of miR-122a target elements were inserted into the 3′-UTR region of HSVtk gene (Fig. 1B) to regulate HSVtk gene in hepatoma cell lines by Ad vectors (20, 21). 

Human hepatoma cell lines, Huh7 and HepG2, expressing different levels of miR-122a (Fig. 2) were chosen as models in this study. The copy number of miRNA binding sites in the 3′-UTR region was based on previously published paper, which showed that almost four copies of miRNA binding sites work better than two or one copies. Insertion of the miR-122a target sequences remarkably decreased the cytotoxicity of HSVtk/GCV in Huh7 cell line (Fig.3); however, no suppression cytotoxicity was observed in the HepG2 cell line. Differences in miRNA expression pattern have been shown to separate transgene expression between intimate cellular lineages; however, the extension of specific applications of these elements to cell and gene therapy will require further tests in suitable experimental models. A main relevance in using the miR-122a-regulated expression system is its potential effect on endogenous targets of miR-122a such as the cationic amino acid transporter-1 gene (17, 22, 23). The effect of miR-122a on levels of endogenous targets of miR-122a were not determined in hepatoma cell lines in this study; however, it is possible that employment of miR-122a to the target sequences incorporated into the HSVtk gene causes a loss of regulation of natural miR-122a targets. Recent studies propose that miRNA-regulated expression of natural targets is not disturbed by large amounts of miRNA target (21). In addition, a feedback loop may be present that increases the expression of miRNAs in response to increasing target elements (22, 24). Further studies will be needed to distinguish this effect. Nevertheless, it is clear that miRNAs can provide a strong method to regulate the expression of a transgene. Here, we demonstrated a model in suicide gene therapy for cancer mediated by Ad vector. In addition, this method has the potential to raise the safety and efficacy of Ad vectors but also as part of other therapeutic strategies.
